# Association Between Opioid Dosage Tapering and Opioid Overdose Among Long-Term Higher-Dose Opioid Users

**DOI:** 10.1016/j.focus.2025.100399

**Published:** 2025-07-29

**Authors:** Hui Zhou, Katherine J. Pak, Fagen Xie, Craig K. Chang, Patricia L. Gray, Deborah S. Ling-Grant, Joanna L. Barreras, Steven G. Steinberg, Rulin C. Hechter

**Affiliations:** 1 Department of Research & Evaluation, Kaiser Permanente Southern California, Pasadena, California; 2 Department of Health Systems Science, Kaiser Permanente Bernard J. Tyson School of Medicine, Pasadena, California; 3 Southern California Permanente Medical Group, Kaiser Permanente, Panorama City, California; 4 Clinical Pharmacy Operations, Kaiser Permanente, Riverside, California

**Keywords:** Opioid, tapering, overdose, electronic health record

## Abstract

**Introduction:**

There has been a steady decline in the national opioid-dispensing rate in recent years. However, emerging evidence that suggests that abrupt discontinuation of opioids for pain may increase overdose risk. The authors examined associations between prescribed opioid tapering and short-term risk of overdose among adults who have received a new long-term high-dose opioid prescription.

**Methods:**

This cohort study included adults (aged ≥18 years) without cancer who were newly prescribed a long-term high-dose opioid, that is, daily dose ≥50 morphine milligram equivalents for ≥183 days, between 2013 and 2018 in Kaiser Permanente Southern California. Opioid tapering within the evaluation period (3 months after being prescribed long-term high-dose opioid) was defined as a reduction ≥10% in monthly morphine milligram equivalents for at least 2 consecutive months compared with baseline morphine milligram equivalents. The association of opioid tapering with incident opioid overdose within 12 months was examined separately using multivariable Cox proportional hazard models with inverse probability of treatment weight. Sensitivity analyses were performed to differentiate the association by tapering rates.

**Results:**

Among 12,866 eligible individuals, 7,372 (57.3%) initiated tapering within 3 months. Seventy-five patients had a documented opioid overdose event within 12 months during follow-up, resulting in an incidence rate of 0.022 and 0.013 event per 1,000 person-years, respectively, among the nontapered and tapered cohorts. Tapering within the evaluation period was associated with a 0.48 (hazard ratio=0.52; 95% CI=0.33, 0.82) reduction in risk of overdose in the subsequent 12 months. Sensitivity analyses suggested that a tapering rate of 20%–40% reduction monthly was associated with lower risk.

**Conclusions:**

Among patients newly prescribed long-term high-dose opioids, initiating tapering within 3 months was shown to be associated with reduced short-term risk of opioid overdose. However, more studies are needed for further confirmation.

## HIGHLIGHTS

• Around 57% of new long-term high-dose opioids users tapered within 3 months.
• The risk for opioid overdose reduced within 12 months after opioids tapering.
• Reducing daily morphine milligram equivalents by 20%–40% monthly was associated with decreased overdoses rate.

## INTRODUCTION

Drug overdose continues to be a devastating public health problem in the U.S. in the past few decades. In 2022, 108,000 overdose deaths were reported by the Centers for Disease Control and Prevention (CDC), and 82,000 of them involved opioids, accounting for about 76% of drug overdose deaths.^1^

Evidence suggests that long-term opioid treatment may not reduce pain significantly for patients without cancer with moderate-to-severe chronic pain.^2^ Instead, long-term opioid exposure has been reported to be associated with elevated risk of multiple adverse effects, including decreased concentration and memory,^3^ changes in mood,^4^ drowsiness,^5^ depression,^6^ and overdose.^7^^,^^8^ In addition, taking opioids at the dosage of 50–99 morphine milligram equivalents (MMEs)/day doubled the risk of overdose death, and the risk was up to 9 times higher if taking ≥100 MME/day than having a dosage <20 MME/day.^9^, ^10^, ^11^ With the increasing concern about the risks and limited therapeutic benefit of long-term opioid therapy among patients without cancer, in 2016, the CDC released a “Guideline for Prescribing Opioids for Chronic Pain,” which delineates appropriate use of long-term opioid therapy and suggests that clinicians consider tapering to a reduced dosage or discontinuing opioid therapy among those using long-term higher doses when the risk of continuing opioids outweighs the benefits; CDC re-enforced this recommendation in 2022.^12^^,^^13^ According to the guideline, a tapering rate between 5% and 20% reduction every 4 weeks is acceptable, and slower tapers such as 10% reduction per month would be appropriate.^13^^,^^14^ After the release of the guideline, prevalence and incidence of long-term opioid use started to decrease after 2016,^15^ and more patients with existing long-term opioid therapy initiated tapering.^16^ However, some studies suggested an increase in potentially opioid-related harms, such as suicide, mental health crises, and overdose in ≥12 months after opioid tapering.^17^, ^18^, ^19^, ^20^ As Fenton et al.^17^ pointed out, a lack of clinical supervision during tapering could have potentially contributed to the increased risk of overdose in these studies. Kaiser Permanente was one of the first health systems in the country to launch a comprehensive, system-wide program to address the problem of prescription opioid misuse. This study sought to provide evidence on the association between opioid dose tapering and overdose from a comprehensive program supporting safe opioid deprescribing in an integrated health system.

## METHODS

### Study Population

The authors conducted a retrospective cohort study of adult members (aged ≥18 years) in Kaiser Permanente Southern California (KPSC), an integrated healthcare system providing comprehensive medical and preventive care to over 4.9 million members in the Southern California service areas. Data on health services received in KPSC facilities are captured through KPSC’s electronic health record (EHR), and care received outside of KPSC facilities is captured through medical insurance claims data. A comprehensive Research Data Warehouse at KPSC is maintained to link administrative and clinical information as well as claims data. The database contains demographic characteristics; linked census tract–level SES; pharmacy dispensing records; and utilization, including diagnoses, procedures, and laboratory results. The pharmacy dispensing records include generic drug name, strength, date of dispensation, quantity dispensed, days’ supply, and National Drug Code (NDC). Mortality information is obtained from National Death Index. The study protocol was reviewed and approved by the KPSC IRB with a waiver for informed consent.

A retrospective cohort of adults (aged ≥18 years) enrolled in KPSC health plans and newly prescribed with long-term high-dose (LTHD) opioids between 2013 and 2018 were identified and followed to examine the association between opioid tapering and risk for opioid overdose. LTHD opioid use was defined following the same identification algorithm as in previous studies.^15^ Specifically, eligible persons were those undergoing a new episode of LTHD opioid use, defined as taking prescribed opioids with a consistent daily dosage ≥50 MMEs for at least 180 days (allowing a maximum gap of 15 days of medication supply on the basis of outpatient pharmacy dispensing records) during the study period. Those with a prior history of being prescribed a LTHD opioid since 2012 were excluded. Patients with a cancer diagnosis or those who were in palliative/hospice care at baseline were excluded. To allow assessment for baseline comorbidities and adequate time for observation of opioid dose tapering, the authors further required ≥12 months prior continuous membership through 3 months after the LTHD opioid use criteria were met.

### Measures

Prescription opioid use was ascertained using identification of NDC in outpatient pharmacy dispensing records. Opioid dosage in MME was calculated using the CDC oral MME conversion factors on the basis of the NDC.^12^^,^^21^ Prescriptions with missing NDC were checked further in the Food and Drug Administration's Drug Code Directory and assigned a conversion factor equal to an exact medication match from the CDC conversion file.^12^^,^^21^^,^^22^ The NDC identifiers were converted to an MME dosage per day (daily MME) with the following calculation: daily MME=MME conversion × dosage × (quantity dispensed/days supplied). For those taking more than 1 opioid medication, the sum of daily doses was calculated by adding up the daily MME calculated for each medication.

Opioid tapering within the 3-month period was ascertained by calculating a moving average daily MME at every 30-day interval for a maximum of 150 days after the date the criteria for LTHD opioid use was met (Appendix Figure 1, available online). The baseline MME was applied from the last monthly MME when the newly prescribed LTHD opioid was initiated or updated to the dose in the 3-month evaluation period if it was higher than the original baseline MME. Tapering episode initiation was defined as a reduction ≥10% in monthly MME for at least 2 consecutive months compared with the baseline MME. The 10% cut off value was chosen on the basis of the “CDC Guideline for Prescribing Opioids for Chronic Pain” published in 2016.^13^ The index date was retrospectively applied to be the first day of reduction once tapering was verified for individuals who initiated tapering within the 3-month baseline period. To avoid the immortal bias, the index date for nontapered patients was randomly assigned to match the distribution of the index date in the tapered population.

For the secondary analysis evaluating the correlation between opioid overdose and tapering rate, the relative percentage of dosage reduction at tapering initiation compared with the baseline dosage among those who initiated tapering within the 3-month baseline period was defined as the tapering rate.

Potential confounders were considered on the basis of clinician recommendations and factors identified in prior literature. Specifically, they include baseline demographic characteristics (age, self-reported sex, race/ethnicity); census tract–level SES, including education attainment and household income; and insurance type (commercial, Medicaid, or Medicare). Baseline clinical factors were identified using the International Classification of Diseases, Ninth Revision (ICD-9) or ICD-10 diagnostic codes as far back as 2 years before the index date, including history of mental health disorders (anxiety disorder, obsessive-compulsive disorder, depression, attention-deficit disorder, disruptive behavior, dementia, eating disorder, bipolar, schizophrenia, personality disorder, and other psychological disorders); history of enrollment in addiction therapy identified by an encounter in the addiction medicine department; history of chronic opioid use defined as having at least 2 continuous opioid therapy episodes with more than 90-days’ supply^15^; substance use disorder; alcohol use disorder; self-inflicted harm; and history of overdose of specific drugs, including opium, heroin, other opioids, methadone, synthetic narcotics, and other/unspecified narcotics. Those without these diagnoses were defined as No for this comorbidity. The Charlson Comorbidity Index was calculated as a proxy for chronic disease burden. History of smoking was self-reported in the EHR.^23^ Concurrent use of benzodiazepines; gabapentinoids; sleep medications, including eszopiclone, zaleplon, and zolpidem tartrate; and/or muscle relaxant drugs at baseline was identified using outpatient pharmacy dispensing records.

The primary outcome included any fatal or nonfatal opioid overdose event within 12 months after the index date. Nonfatal overdoses were identified by ICD-9/ICD-10 diagnostic codes recorded in any emergency visit or inpatient setting, and fatal overdoses were identified by records of ICD-10 codes from National Death Index records during follow-up (Appendix Table 1, available online). All patients were followed from their index date to the first occurrence of an opioid overdose event within 12 months. People were censored after membership disenrollment, death due to other causes, end of 12 months, and administrative end of the study period (June 30, 2020), whichever occurred first.

### Statistical Analysis

The distribution of baseline demographic, socioeconomic, and clinical characteristics among individuals who initiated opioid tapering was compared with that among those who did not initiate tapering. To avoid the limitation of using *p*-values with large sample sizes, the statistical significance of differences between the 2 groups was evaluated by effect size (absolute standardized mean difference) using Cohen’s D.

To account for potential confounding and selection bias, the inverse probability of treatment weight (IPTW) was used in the modeling. Briefly, a propensity score was created using logistic regression to represent the possibility of tapering initiation within the 3-month baseline period for individuals without a prior history of opioid use and who were newly prescribed LTHD opioids. The logistic model included baseline demographic characteristics (age, sex, race/ethnicity); census tract–level SES (education, income); and other factors based on literature review and clinical knowledge, such as baseline average daily MME, Charlson Comorbidity Index, history of mental health disorders, history of substance use disorders and smoking, concurrent use of sleep medications, muscle relaxant drugs, benzodiazepines, or gabapentinoids, and index year.^24^ The stabilized inverse propensity score was then applied as a weight to create a pseudostudy population for the analysis. Absolute standardized mean difference was calculated to examine whether the potential confounders were balanced between the comparison groups after applying the IPTW. A Cohen’s D <0.1 was considered to indicate a negligible difference.^25^ To estimate the association between tapering and opioid overdose (fatal or nonfatal) in 12 months, Cox proportional hazard models were applied to the balanced pseudopopulation to obtain the hazard ratio (HR) and its 95% CI.

A secondary analysis was performed among the same study population to further assess the risk of fatal and nonfatal opioid overdose associated with different tapering rates, including discontinuation, compared with that among those who did not initiate tapering within 3 months. All statistical analyses were conducted using the SAS Enterprise Guide (Version 8.2, SAS Institute Cary, GA).

## RESULTS

**Figure 1:**
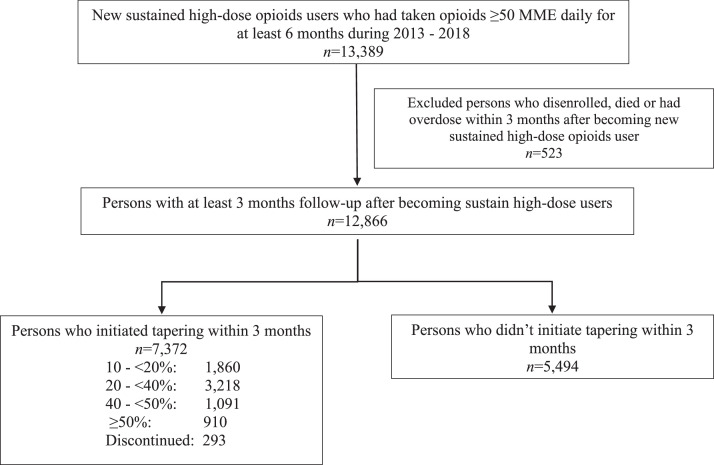
Study cohort establishment. MME, morphine milligram equivalent.

A total of 12,866 individuals who were newly prescribed LTHD opioids were included in the analytical sample after exclusion criteria were applied (Figure 1). Among those, 7,372 (57.3%) individuals initiated opioid tapering, with a subset of 293 (2.3%) persons who discontinued opioid use completely within the 3-month evaluation period. Among those who initiated tapering, 4,310 (58.5%) individuals began tapering within 1 month; 1,702 (23.1%) started in the second month; and 1,360 (18.5%) initiated in the third month after they met the criteria for LTHD opioid use.

The distributions of baseline demographic characteristics, census tract–level SES, and selected clinical conditions are presented in Table 1. Individuals who initiated tapering were older, less likely to be of non-Hispanic White race, and less likely to be a current smoker than those who did not initiate tapering within 3 months, but the absolute standardized mean difference of these characteristics was less than 0.1, indicating a negligible difference (Appendix Figure 2, available online). Those initiating tapering were more likely to have a higher average daily MME at baseline and a history of chronic opioid use for more than 90 days, for which the absolute standardized mean difference was greater than 0.1, indicating a small difference between 2 groups. These differences were negligible after IPTW.

**Table 1:** Baseline Characteristics Comparison Among Patients With Newly Prescribed Long-Term High-Dose Opioids With and Without Tapering Initiation Within 3 Months

Characteristics	No tapering initiation within 3 months	Tapering initiated within 3 months
	*n*=5,494	*n*=7,372
Age at index, *n* (%), years		
18–33	409 (7.4)	548 (7.4)
34–49	1,447 (26.3)	1,870 (25.4)
50–64	2,356 (42.9)	3,039 (41.2)
≥65	1,282 (23.3)	1,915 (26.0)
Female, *n* (%)	3,062 (55.7)	4,231 (57.4)
Race/ethnicity, *n* (%)		
Non-Hispanic White	3,543 (64.5)	4,517 (61.3)
Hispanic	1,189 (21.6)	1,742 (23.6)
Non-Hispanic Black	560 (10.2)	856 (11.6)
Asian	80 (1.5)	116 (1.6)
Others	122 (2.2)	141 (1.9)
Medicaid at index date, *n* (%)	1,320 (24.0)	1,837 (24.9)
Median household income, *n* (%)		
≤$30,000	227 (4.1)	338 (4.6)
$30,001–$60,000	2,549 (46.4)	3,512 (47.6)
$60,001–$100,000	2,281 (41.5)	2,914 (39.5)
>$100,000	422 (7.7)	574 (7.8)
Missing	15 (0.3)	34 (0.5)
High-school graduate and above, *n* (%)		
0%–50%	116 (2.1)	205 (2.8)
51%–75%	1,341 (24.4)	1,938 (26.3)
76%–100%	4,022 (73.2)	5,195 (70.5)
Missing	15 (0.3)	34 (0.5)
Charlson Weighted Comorbidity, *n* (%)		
0	2,099 (38.2)	2,722 (36.9)
1–2	2,168 (39.5)	2,858 (38.8)
≥3	1,227 (22.3)	1,792 (24.3)
Mental health disorders, *n* (%)	4,129 (75.2)	5,496 (74.6)
Anxiety disorder	2,457 (44.7)	3,259 (44.2)
Obsessive compulsive disorder	29 (0.5)	53 (0.7)
Depression	2,456 (44.7)	3,427 (46.5)
Substance abuse	2,097 (38.2)	2,732 (37.1)
Attention deficit disorder	168 (3.1)	218 (3.0)
Disruptive behavior disorder	2 (0.0)	6 (0.1)
Dementia	135 (2.5)	216 (2.9)
Eating disorder	71 (1.3)	126 (1.7)
Bipolar disorder	241 (4.4)	359 (4.9)
Schizophrenia	51 (0.9)	75 (1.0)
Other psychosis	71 (1.3)	101 (1.4)
Self-inflicted injury	116 (2.1)	167 (2.3)
Personality disorder	74 (1.3)	114 (1.5)
Smoking status, *n* (%)		
Current	1,178 (21.4)	1,404 (19.0)
Former	1,790 (32.6)	2,439 (33.1)
Never	2,117 (38.5)	3,050 (41.4)
Unknown	409 (7.4)	479 (6.5)
History of substance abuse		
Nicotine dependence	1,289 (23.5)	1,665 (22.6)
Alcohol	283 (5.2)	400 (5.4)
Other drugs	484 (8.8)	722 (9.8)
History of overdose, *n* (%)	43 (0.8)	50 (0.7)
Enrollment in addiction therapy^a^, *n* (%)	291 (5.3)	465 (6.3)
MME before tapering, median (IQR)	69.0 (60.0, 98.0)	87.0 (61.5, 128.0)
Co-use of medications at baseline^b^, *n* (%)		
Benzodiazapine	1,453 (26.4)	2,011 (27.3)
Gabapentinoid	1,107 (20.1)	1,316 (17.9)
Sleeper	368 (6.7)	512 (6.9)
Muscle relaxant	1,201 (21.9)	1,504 (20.4)
History of chronic opioid use^c^		
<1 year	3.629 (66.1)	5,311 (72.0)
1–2 years	1,019 (18.6)	1,204 (16.3)
≥2 years	846 (15.4)	857 (11.6)

a Enrollment in addiction therapy was defined as those who had an encounter in the addiction medicine department within 2 years prior to index date.

b Co-use of benzodiazepine, gabapentinoids, sleeper medications, or muscle relaxants with an overlap of opioid use for at least 30 days.

c History of chronic opioid use is defined as the duration of continuous opioid dispense allowing 15-day gap leading up to index date looking back 5 years. MME, morphine milligram equivalent.

During follow-up, 75 individuals had at least 1 opioid overdose event, including 13 fatal overdoses, within 12 months after the index date (Table 2). In the balanced pseudopopulation after IPTW (Appendix Figure 2), tapering initiation within 3 months was associated with a statistically significant risk of opioid overdose within 12 months (HR=0.52; 95% CI=0.33, 0.82) (Table 2).

**Table 2:** Hazard Ratio of Opioid-Related Overdose (Fatal or Nonfatal) Within 12 Months Between Patients With Newly Prescribed Long-Term High-Dose Opioids With and Without Tapering Initiation Within 3 Months

Group	Overdose event within 12 months			
	Number of patients	Number of event	Incident rate (*n*/1,000 person-years)	HR (95% CI)
No tapering initiation within 3 months	5,494	42	0.0218	ref
Tapering initiation within 3 months	7,372	33	0.0129	0.52 (0.33, 0.82)
10% to <20%	1,860	10	0.0154	0.61 (0.31, 1.23)
20% to <40%	3,218	12	0.0107	0.40 (0.21, 0.78)
40% to <50%	1,091	4	0.0105	0.43 (0.15, 1.23)
≥50%	1,203	7	0.0172	0.77 (0.35, 1.70)

Among 7,372 patients who initiated tapering, 903 (12.2%) started tapering with a dosage reduction rate ≥50%, and 290 (4.0%) discontinued opioids within 3 months. In the secondary analysis examining the association between tapering rate and the risk for opioid overdose, an initial tapering rate between 20% and 40% appeared to be associated with a reduced risk for opioid overdose (HR=0.40; 95% CI=0.21, 0.78) (Table 2). A trend appeared showing reduced risk of overdose within 12 months with any tapering initiated within 3 months after meeting criteria for long-term higher-dose opioid use.

## DISCUSSION

In this large retrospective cohort study of adults newly prescribed LTHD opioids in an integrated healthcare system, initiation of opioid dosage tapering within 3 months was associated with reduced risk of opioid overdose during the 12-month follow-up period.

Previous studies have raised concern that dosage tapering among those using opioids for the long term could experience negative effects related to uncontrolled or undercontrolled pain. No study, to the authors’ knowledge, reported clinically significant worsening of pain after tapering.^26^^,^^27^ However, several studies reported an elevated risk of overdose or mental health crises up to 4 years after tapering initiation. In those studies, anyone who received long-term opioid therapy (either ≥90 daily MME for more than 90 days)^20^ or ≥90 days with ≥90% days on therapy^28^ or prescribed a stable higher dose of opioids (≥50 MME daily for 12 months) were included, mixed with prevalent and newly initiated long-term opioid use. Because the motivation for tapering was potentially different between prevalent and incident LTHD opioid users, the risk of overdose could also differ. In this study, the authors focused on those who were newly prescribed with LTHD opioids because their initiative for tapering and behaviors were likely different from those of persons who have been using higher-dose opioids for an extended time, which subsequently may result in disparate risk of overdose. The results show a reduced risk of overdose associated with tapering, which is consistent with other studies, including an RCT by Sullivan et al.,^29^ retrospective cohort studies performed in Canada^28^ and the U.S.,^30^ and a large study of commercially insured individuals who received long-term opioid treatment.^31^ This finding aligns with the Food and Drug Administration safety announcement published on April 9, 2019^19^ and consistent with those of other studies.^28^^,^^32^ Guidelines from the CDC and Department of Veterans Affairs/Department of Defense suggest that gradual dose tapering over months to years is associated with a lower rate of healthcare utilization owing to opioid-related adverse events than abrupt discontinuation or tapering in less than 3 weeks.^33^^,^^34^ One other study also suggested a negative relationship between the speed of dose reduction and withdrawal symptoms.^35^

Many studies to date support complementary approaches such as psychological support and cognitive behavioral therapy in contributing to the reduction of opioid dose without increasing pain severity. Kaiser Permanente had a multifaceted program to address the problem of prescription opioid misuse. Therefore, in contrast to the increased risk of overdose reported shortly after tapering in other study settings,^17^^,^^18^^,^^28^ this study showed decreased risk of opioid overdose associated with tapering. As Dowell and colleagues^12^ stated, additional research studies are needed to build the evidence base for optimal pain management and to increase the “effectiveness of clinician and health system strategies to promote equitable access to high-quality pain management.” This study filled in the blank by providing evidence that a comprehensive program supporting safe opioid deprescribing in an integrated health system can assist patients even before they make the decision to enter the process of tapering and potentially reduce opioid overdose risk.

### Limitations

This study has several limitations. First, the use of outpatient opioid-dispensing records can most reliably identify prescription opioid use within KPSC; however, nonprescribed drug use obtained outside of KPSC was not recorded. Second, KPSC members receive care with prepaid health insurance and are slightly different in composition from the general population, including higher percentages of Hispanic, Latino, and Asian people; higher education level completed; and higher household income. Therefore, the findings may not be generalizable to people without insurance or who are underinsured or with lower SES.^36^ Not only the socioeconomical status differs, but there is the potential difference in the care that members can receive because Kaiser Permanente’s practice pattern combines health plan coverage with medical services, whereas other health systems do not.^37^ Third, using ICD-9/ICD-10 codes to identify opioid overdose events including fatal overdose from death certification can potentially misclassify the cause and miss some cases. Fourth, the authors did not assess the potential impact of the coronavirus disease 2019 (COVID-19) pandemic on overdose diagnosis. Fifth, the current analysis was intent to treat, where tapering rate was determined by months 7–9 after having 6 months of LTHD opioid use, and it was assumed that tapering status remained stable afterward, although results may likely differ if the follow-up time varies. Finally, although the authors tried to balance potential known confounders in modeling using IPTW, unmeasured confounders may potentially influence the results, such as types of pain diagnoses and nonpharmacologic pain treatments implemented prior to tapering.

## CONCLUSIONS

In this retrospective cohort study using EHR data from a large, diverse, insured population, the authors found that patients who initiated tapering within the first 3 months after starting a new prescription of LTHD opioids showed a reduced risk of overdose within 12 months compared with those who did not taper. More studies in this area are needed to better understand this association.

## Appendix Supplementary materials

Image, application 1

## Footnotes

Supplementary material associated with this article can be found in the online version at doi:10.1016/j.focus.2025.100399.
