# Supplementary material for: Association Between Opioid Dosage Tapering and Opioid Overdose Among Long-Term Higher-Dose Opioid Users
Source: AJPM Focus. 2025 Jul 29;4(6):100399. doi: 10.1016/j.focus.2025.100399 (PMC12508818; doi:10.1016/j.focus.2025.100399)
Supplement: Supplementary file 1 [file mmc1.docx]

**Appendix**

**Appendix Table 1.** Opioid-related overdose dx codes

|  | **ICD-9** | **ICD-10** |
| --- | --- | --- |
| Non-Fatal | 965.00, 965.01, 965.09, 965.02, E850.0, E850.1, E850.2, E935.2 | T40.0X1A, T40.0X2A, T40.0X4A, T40.0X5A,  T40.1X1A, T40.1X2A, T40.1X4A,  T40.2X1A, T40.2X2A, T40.2X4A, T40.2X5A, T40.2X6A,  T40.3X1A, T40.3X2A, T40.3X4A, T40.3X5A, T40.3X6A,  T40.4X1A, T40.4X2A, T40.4X4A, T40.4X5A, T40.4X6A,  T40.601A, T40.602A, T40.603A, T40.604A, T40.605A, T40.606A, T40.691A, T40.695A |
| Fatal | N/A | T40.0, T40.1, T40.2, T40.3, T40.4, T40.6 |

New patients who were prescribed long-term high-dose opioid with ≥ 50 daily MME for at least 180 days

At least two consecutive months with daily MME reduction ≥10% from previous highest dose or discontinued for ≥ 30 days

**Evaluation period**

**Appendix Figure 1.** Algorithms of identifying long-term higher-dose opioids users and evaluating tapering initiation

**Appendix Figure 2**. Absolute standardized mean difference before and after IPTW between patients with newly prescribed long-term high-dose opioids who did and did not initiate tapering within 3 months.
